# Surgical management of green nail syndrome

**DOI:** 10.1016/j.jdin.2025.12.003

**Published:** 2025-12-19

**Authors:** Joong Heon Suh, Jungyoon Ohn, Keunyoung Hur, Je-Ho Mun

**Affiliations:** a Department of Dermatology, Seoul National University Hospital, Seoul, Republic of Korea; b Department of Dermatology, Seoul National University College of Medicine, Seoul, Republic of Korea; c Institute of Human-Environment Interface Biology, Seoul National University Medical Research Center, Seoul, Republic of Korea

**Keywords:** green nail syndrome, nails, nail diseases, patient case management, *Pseudomonas aeruginosa*, surgery, therapeutics

*To the Editor:* Green nail syndrome (GNS) is an infectious nail disease commonly associated with *Pseudomonas aeruginosa*, characterized by greenish pigmentation of the nail. Untreated cases may lead to infections in various organs, including pseudomonal keratitis with persistent corneal scarring.^1^ Medical treatment, including topical or systemic antibiotics, is considered the first-line therapy. However, surgical intervention may be warranted in some cases because of the substantial bacterial burden on the nails and associated nail dystrophy.^2^^,^^3^ Here, we report a series of cases of GNS that were successfully treated surgically at our institution.

We retrospectively reviewed cases diagnosed with GNS who underwent surgical treatment at the Department of Dermatology, Seoul National University Hospital, between January 2015 and March 2025. Inclusion criteria were severe GNS (>50% nail surface involvement) with greenish color and nail dystrophy, surgical treatment performed, and available follow-up data. All cases meeting the inclusion criteria during the study period were included. No cases were excluded based on treatment response.

Thirteen patients (8 men and 5 women) were included and their clinical characteristics are summarized in Table I. Bacterial cultures identified microorganisms in all cases (100%), including *P. aeruginosa* in 12 patients (92%). Fungal coinfection was identified in 9 patients (69%) (Supplementary Fig 1, available via Mendeley at https://data.mendeley.com/datasets/4nnvnd27sy/1). Patients presented various nail dystrophies, such as nail plate thickening, onycholysis, shrimp-shaped nail change, onychomadesis, and retronychia (Supplementary Fig 2, available via Mendeley at https://data.mendeley.com/datasets/4nnvnd27sy/1).

**Table I:** Clinical characteristics of patients with green nail syndrome

Case number	Clinical characteristics										
	Demographics			Locations		Microorganisms		Surgery	Medication	Prognosis	
	Sex	Age	Onset	Toe or finger	Rt/Lt/Bt xth	Bacterial culture results	Fungus culture results and/or special stain	Type	Perioperative antimicrobial/antifungal therapy	Follw-up duration (Mo)	Improvement
1	F	47	1 y	Toe	Bt 1st	*P. aeruginosa* *C. freundii* *Vagococcus species*	No fungus	Total avulsion		102	Clear
2	F	49	6 mo	Toe	Rt 1st	*P. aeruginosa* *E. coli* *E. Casseliflavus*	GMS, PAS: Fungal hyphae and spores	Total avulsion	Terbinafine 250 mg × 7 wk (Post)	12	Clear
3	F	53	N/A	Toe	Lt 1st	*E. faecalis* *S. maltophilia*	GMS, PAS: Fungal hyphae and spores	Total avulsion	Ciprofloxacin 750 mg × 3 wk (Pre)	113	Clear
4	M	50	2 mo	Toe	Bt 1st	*P. aeruginosa* *S. marcescens*	No fungus	Total avulsion		76	Clear
5	M	16	2 y	Toe	Bt 1st, Lt 2nd	*P. aeruginosa*	No fungus	Total avulsion		60	Clear
6	M	81	1 m	Toe	Bt 1st	*P. aeruginosa* *S. maltophilia*	GMS: Fungal hyphae and spores	Total avulsion	Ciprofloxacin 500 mg × 7d (Post)	9	Clear
7	F	38	2 mo	Toe	Lt 1st	*P. aeruginosa* *E. gergoviae*	No fungus	Partial avulsion		79	Clear
8	M	40	4 y	Toe	Rt 1st	*P. aeruginosa* *S. marcescens*	GMS, PAS: Fungal hyphae and spores *Culture: T. rubrum*	Partial avulsion		108	Clear
9	F	55	3 m	Toe	Rt 1st	*P. aeruginosa* *E. faecalis*	GMS, PAS: Fungal hyphae and spores	Nail plate excision of the involved portion	Itraconazole 400 mg for 1 wk x 4 cycles (Post)	11	Clear
10	M	32	1 mo (Nail dystrophy: 1 y)	Toe	Rt 1st	*P. aeruginosa* *E. casseliflavus*	GMS, PAS: Fungal hyphae and spores	Nail plate excision of the involved portion	Ciprofloxacin 500 mg × 7 d (Post), Terbinafine 250 mg × 3 mo (Post)	101	Clear
11	M	70	7 mo	Toe	Rt 1st	*P. aeruginosa* *E. faecalis*	GMS, PAS: Fungal hyphae and spores	Nail plate excision of the involved portion	Itraconazole 200 mg × 10 wk (Post)	98	Clear
12	M	65	6 mo	Toe	Rt 1st	*P. aeruginosa* *E. casseliflavus* *C. Koseri*	GMS, PAS: Fungal hyphae and spores *Culture: T. rubrum*	Nail plate excision of the involved portion	Terbinafine 250 mg × 3 mo (Post)	92	Clear
13	M	59	2 mo	Toe	Lt 1st	*P. aeruginosa* *S. marcescens*	GMS, PAS: Fungal hyphae and spores	Nail plate excision of the involved portion	Terbinafine 250 mg × 3 mo (Post), Topical Ofloxacin x 1 wk (Post)	28	Clear

*A. Materials,* Adherent materials; *Bt*, both; *GMS*, Grocott’s methenamine silver stain; *C. freundii*, Citrobacter freundii; *C. koseri*, Citrobacter koseri; *E. casseliflavus*, Enterococcus casseliflavus; *E. coli*, Escherichia coli; *E. faecalis*, Enterococcus faecalis; *E. gergoviae*, Enterococcus gergoviae; *P. aeruginosa*, Pseudomonas aeruginosa; *PAS,* Periodic acid-Schiff stain; *S. maltophilia*, Stenotrophomonas maltophilia; *S. marcescens*, Serratia marcescens; *T. rubrum*, Trichophyton rubrum.

Following disinfection and local anesthesia, the involved nail plates were removed using a free septum elevator and double-action nail nipper (Supplementary Figs 3 and 4, available via Mendeley at https://data.mendeley.com/datasets/4nnvnd27sy/1). Depending on the extent of nail involvement and dystrophies, total nail avulsion, partial nail avulsion, and nail plate cutting of the involved portion preserving the proximal nail plate were performed in 6 (50%), 2 (16.7%), and 4 patients (33.3%), respectively (Supplementary Fig 5, available via Mendeley at https://data.mendeley.com/datasets/4nnvnd27sy/1). After the procedure, all cases were prescribed topical antibiotics, including mupirocin (12 patients) and ofloxacin (1 case), for postoperative dressing. Among the patients, oral antipseudomonal therapy was used in 3 cases (2 postoperative), and antifungal therapy in 7 cases, as shown in Table I. All patients showed complete resolution of the GNS (Fig 1, Supplementary Fig 4, available via Mendeley at https://data.mendeley.com/datasets/4nnvnd27sy/1).

**Fig 1:**
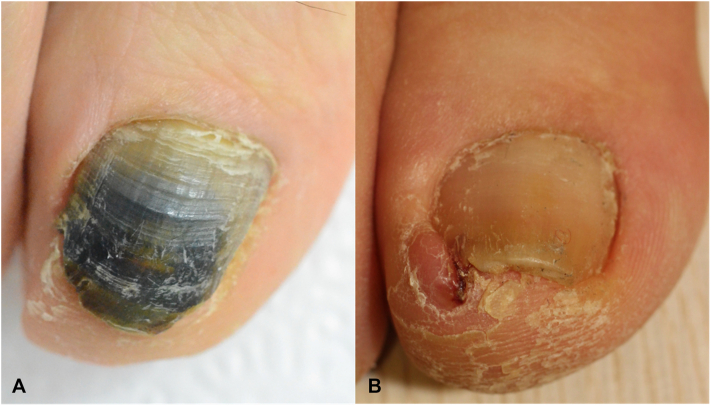
*Green* nail syndrome of the great toenail before **(A)** and 6 months after surgical total nail avulsion **(B)**. Marked improvement is observed in *green* discoloration and associated nail dystrophy (images of case 2 and surgical procedure is detailed in Supplementary Fig 3, available via Mendeley at https://data.mendeley.com/preview/4nnvnd27sy).

Antibiotic treatment remains the primary treatment for GNS; however, its efficacy in severe GNS remains unclear owing to the absence of randomized controlled trials. Medical treatments are often inadequate in extensive or chronic cases, particularly in those involving nail dystrophies or concomitant fungal infection.^4^

Data from our tertiary referral center revealed that in a subset of GNS cases, adherent material with a foul odor was frequently observed between the nail plate and bed. *P. aeruginosa* forms biofilms that impair antibiotic efficacy and evade host immune clearance.^5^ Therefore, we presume that the adherent material, likely a biofilm, contributes to the resistance to medical treatment and development of nail dystrophies. In such cases, surgical removal of the nail plate and adherent material appears to be an appropriate treatment. Surgical avulsion of the dystrophic nail resulted in improvement of the GNS and associated dystrophic nail changes (Fig 1).

Overall, we suggest that surgical intervention is an effective treatment option for GNS, particularly in severe and refractory cases. A limitation of this study is its single-center retrospective design. Future randomized controlled trials are needed to clarify the indications and comparative efficacy of medical versus surgical management of mild and severe GNS.

## Conflicts of interest

None disclosed.
